# Testing ethical disagreement on ancestral human remains in museums

**DOI:** 10.1017/S094073912610023X

**Published:** 2026-02-11

**Authors:** Errol Francis, Chloe Asker, Victoria Tischler

**Affiliations:** 1 Culture&, United Kingdom; 2 School of Psychology, University of Surrey, United Kingdom

**Keywords:** ethical disagreement, ancestral remains, human remains, museums, engagement, participation

## Abstract

The display of ancestral human remains in museums is a contentious ethical issue, raising concerns around the dignity and respect for ancestral lived lives versus the role of remains for education and scientific enquiry. Against the backdrop of recent debates sparked by the deinstallation of ancestral remains at several museums (e.g., the removal of the Shuar tsantsas at the Pitt Rivers Museum) and revisions of national and international ethics codes, this essay explores the role of two methodologies – a trial and interactive workshop – in producing inclusive spaces to support ethical decision making and practice. Digital participation technologies were used to support an accessible mode of participation that was anonymous – allowing attendees to express opinions about emotive and challenging subjects, such as ancestral human remains. For both examples, attendees and participants identified key priority and action areas for the sector and within their places of work. The activities will contribute to a wider research project that is investigating value and ethical disagreements and polarization within museums.

## Introduction

This paper explores two participative, inclusive, and interactive methods of engaging stakeholders with ethical issues in the museum sector, focusing on disagreements about the display of human remains. This work is set against a background of increasing unease about the ethics of displaying human remains in museums.

## Background

Human remains are located on a spectrum, existing somewhere between objects of science and lived lives, depending on the ways in which they are encountered, framed, and understood.^1^ As scientific specimens, they cater for the education of visitors, often becoming subject to fascination and exoticization as exhibited bodies. On the other hand, these remains, as ancestral lived lives, are worthy of greater dignity and respect and, in many cases, repatriation back to living ancestors. This essay uses the terms “ancestral remains” and “human remains” interchangeably, depending on the legislative, social, political, and cultural context; however, these terms have different meanings.

National Galleries Scotland refers to human remains as “the bodies, and parts of bodies, of members of the species *Homo sapiens*. This includes osteological material (whole or part skeletons, individual bones or fragments of bones and teeth), soft tissue including organs, skin, cornea, bone marrow, embryos, and slide preparations of human tissue, nails, and hair. It is acknowledged that some cultural communities give these a sacred importance.”^2^ On the other hand, the term “ancestral remains” does not just refer to human body parts kept and displayed in museum collections; rather it attempts to confer a sense of familial belonging and individuality to the biological assets of collected persons. “Ancestral remains” refers to multilinear cultural relationships to living contemporary communities who may or may not be biogenetically related in terms of inherited DNA. It is a reminder to us of the connections between the human past, the present, and the personhood of these collected items – “deeply connected with the collective soul of the community to which they belong.”^3^ The authors of the article adhere to this complex and culturally situated understanding of ancestral remains.

In September 2020, following a three-year ethical review, the Pitt Rivers Museum at the University of Oxford removed one of its most popular displays. The Shuar *tsantsas*, otherwise referred to as “shrunken heads,” have been on display at the museum for many years in a vitrine entitled “The Treatment of Dead Enemies.” The ancestral remains are regarded as sacred by the Shuar and Achuar peoples of Ecuador, from where they originated and were acquired by Western collectors, and their removal from display by the museum sparked conflicting public debate.^4^ In addition to the tsantsas, the museum has also removed South Asian Naga trophy heads and an Egyptian mummy of a child from open display.^5^

In 1904, a game hunter, James Harrison, brought to Britian two women and four men of the Bambuti ethnic group (who live in the Ituri Forest in what is now the Democratic Republic of Congo, Central Africa).^6^ The Bambuti group were displayed in various British cities as part of a tour that many now describe as a “human zoo,” and they were viewed by over a million people. In 1906, Amuriape, one of the Bambuti women, gave birth to a girl in Bedford who was stillborn. The remains of a stillborn Bambuti baby ended up in the collection of the Hunterian Museum in London and until recently were listed in the museum’s catalogue of items that can be viewed for medical research. However, in 2024, after criticism by writer and filmmaker Nadifa Mohamed, the remains of the baby were removed from “display.”^7^

Against this background, leading UK sector body the Museums Association has updated its code of ethics in 2025.^8^ Since the beginning of 2024, the association has engaged in an extensive consultation to update its Code of Ethics,^9^ with the question of ancestral remains being an important aspect of the review. The code includes two direct references to human remains:

1.8 Be understanding of different religious, spiritual, and cultural perspectives when working with collections, particularly human remains and sacred items.^10^

2.22 Be clear and open about why the museum holds human remains.^11^

The code (2.23) also states a clear commitment to an “open, proactive and positive approach to repatriation and restitution.”^12^

Similarly, in 2019, the International Council of Museums (ICOM) at their 25th General Conference decided to review and revise their code of ethics. ICOM sets minimum professional standards and encourages the recognition of values shared by the museum community internationally. The review process began in 2022, involving four consultations, and the revised code will be presented for review at the ICOM Annual Meeting in June 2026.^13^ The 2017 code of ethics treats human remains as culturally sensitive material of sacred significance (2.5, p. 10).^14^ Their acquisition should only be granted if the remains can be housed securely, cared for respectfully (3.7, p. 20), and displayed with “great tact and respect for the feelings of human dignity” (4.3, p. 25) – consistent with professional standards and beliefs from community, ethnic, or religious groups from whom the objects originated. Requests for removal should be “addressed expeditiously with respect and sensitivity” (4.4, p.25).

In contrast, a report by the UK Parliament’s UK All-Party Parliamentary Group for Afrikan Reparations (APPG-AR) argues for radical changes to the legalization, research, and return of ancestral remains in the United Kingdom. The report recommends that it should be an offence to sell ancestral remains or publicly display them without consent,^15^ calling for the Human Tissue Act 2004, which covers England, Wales, and Northern Ireland, to be amended to make it an offence to publicly display any human remains, irrespective of age or provenance, without “appropriate consent”; and further amending the act to require museums and other institutions to obtain a licence for any human remains held in their collections – a requirement that presently applies only to human remains dated to within the past 100 years. Furthermore, the report identifies that more funding is required to map the inventory of ancestral remains in museums and cultural organizations across the United Kingdom. The APPG-AR’s approach foregrounds the voices of civil society and community groups through recommending that African and African diasporic communities, organizations, and movements can participate in the debates regarding restitution and repatriation of ancestral remains.^16^

Concerns have been raised regarding the recommendations the APPG-AR report authors. In May 2025, the British Association for Biological Anthropology and Osteoarchaeology (BABAO) issued a joint statement responding directly to the APPG’s recommendations. While the group supports legislative change to prohibit the sale of human remains, BABAO criticises the use of the term “ancestral remains,” remarking on its ambiguity regarding genealogical descendancy, and argues that the “proposed wording would have far-reaching and potentially unintended consequences for the study, management, care, and public engagement with all … archeologically derived remains irrespective of provenance.”^17^ BABAO expresses their concern within the context of the revolution in archaeological methods, which has given rise to the demand for access to human biomaterials.^18^

Museums are positioned within this divide, on one side tasked with curation, conservation, and repatriation, and on the other facing demands for access to and retention of collections for research and education.^19^ While many institutions are repatriating remains of both humans and animals, for example the recent return of ancestral human remains to Hawaii following a formal request to the Ulster Museum (Northern Ireland) from the Office of Hawaiian Affairs,^20^ others are arguing that displaying and using remains as educational resources and subjects for DNA research is ethical and the best way to respectfully honor the deceased.^21^ However, as Joyce points out in her work on science and objectivity, while arguments against the repatriation of human remains are legitimized by spurious reasons associated with academic and scientific freedom, “scientists do not actually have the right to conduct research on any topic in any way that they wish.”^22^ Research, as the production of knowledge (and not truth), must be responsible and ethical – taking into account various ethical considerations, guidelines, permitting processes, and reviews.

It is in the context of these polarized debates^23^ that this short essay emerges and is situated within a larger research project, ANTITHESES, that is part of the Wellcome Discovery Research Platform for Transformative Inclusivity in Ethics and Humanities Research at the University of Oxford.^24^ The research seeks to investigate the site of the museum as a locale that is at the heart of ethical disputes, which reflect urgent sociopolitical and cultural issues. In this essay, we discuss two examples of interventions that aimed to support ethical decision making and practice. Example 1 focuses on the process of undertaking an intervention at University of Leicester’s Research Centre for Museums and Galleries (RCMG) to explore the ethical disputes around ancestral remains. Example 2 explores the activities at the Museums Association (MA) 2024 Conference which raised questions regarding professional ethical practices.

## The New Museum School Advanced Programme Symposium

On May 29–30, 2024, at the New Museum School Advanced Programme (NMSAP) Symposium that took place at the University of Leicester, students and staff had the opportunity to engage more extensively with a range of ethical questions related to ancestral remains. The program is part of a joint initiative led by Culture& with RCMG to provide diverse pathways into museums studies for underrepresented groups. Culture& is an independent arts and education charity based in London, working for nearly 30 years in partnership with arts and heritage institutions and artists to develop programs that aim to open up the arts and heritage workforce through workforce training and aligned public programs.

Culture& provide fully funded studentships for postgraduate diploma and masters programs in museum studies and socially engaged practice in museums and galleries to UK-based individuals from backgrounds underrepresented in the museum and heritage sector, particularly those from the global majority. NMSAP is supported by the Esmee Fairburn Foundation, The Marstine Family Foundation, and the Art Fund.^25^ The program supports diverse talent with leadership potential to benefit from a gold standard postgraduate offer; develop their careers within museums, galleries, and heritage (through links to established UK cultural parters, e.g., Wellcome Collection and the Pitt Rivers Museum, University of Oxford); and support the inclusive transformation of the sector. Over 150 people from diverse backgrounds have received this training so far, with around 74% finding full-time employment in the sector within six months of graduating^26^. The NMSAP Symposium takes place every year in the autumn and explores novel approaches to socially engaged practice in arts, heritage, and cultural institutions.

At the 2024 symposium, students gathered with academics, staff from cultural and heritage organizations, and other stakeholders at the event. The symposium provided an opportunity for students to reflect on their studies, to receive specialist input on topics of sector concern such as ethically interpreting traumatic collections, and to engage in networking activities. This symposium focused on ethical disagreements around the acquisition and display of ancestral human remains and used an engaging and performative methodology – in the form of a trial – to contrast polarized perspectives and debates.

## The Trial

The second day of the symposium featured a simulated trial considering issues associated with collecting, storing, and displaying ancestral remains. Attendees were invited to participate in the trial by “voting,” namely responding to questions via an online digital voting app (AhaSlides). This collated responses and provided summary results in visual form that were presented live at the symposium and are summarized subsequently.

**Figure 1:**
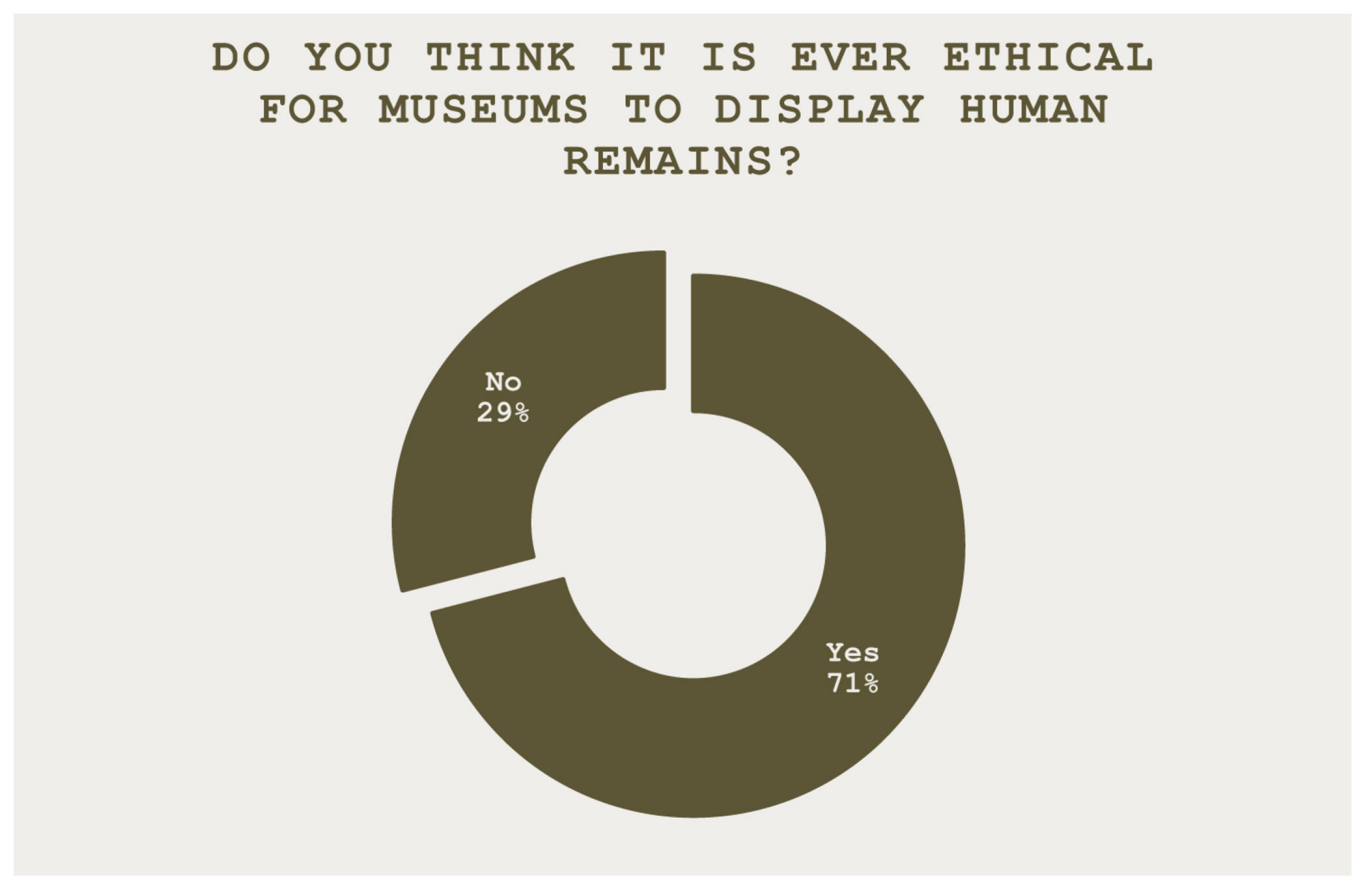
Do you think it is ever ethical for museums to display human remains? Twenty-two attendees (71%) voted yes, and nine (29%) voted no.

**Figure 2:**
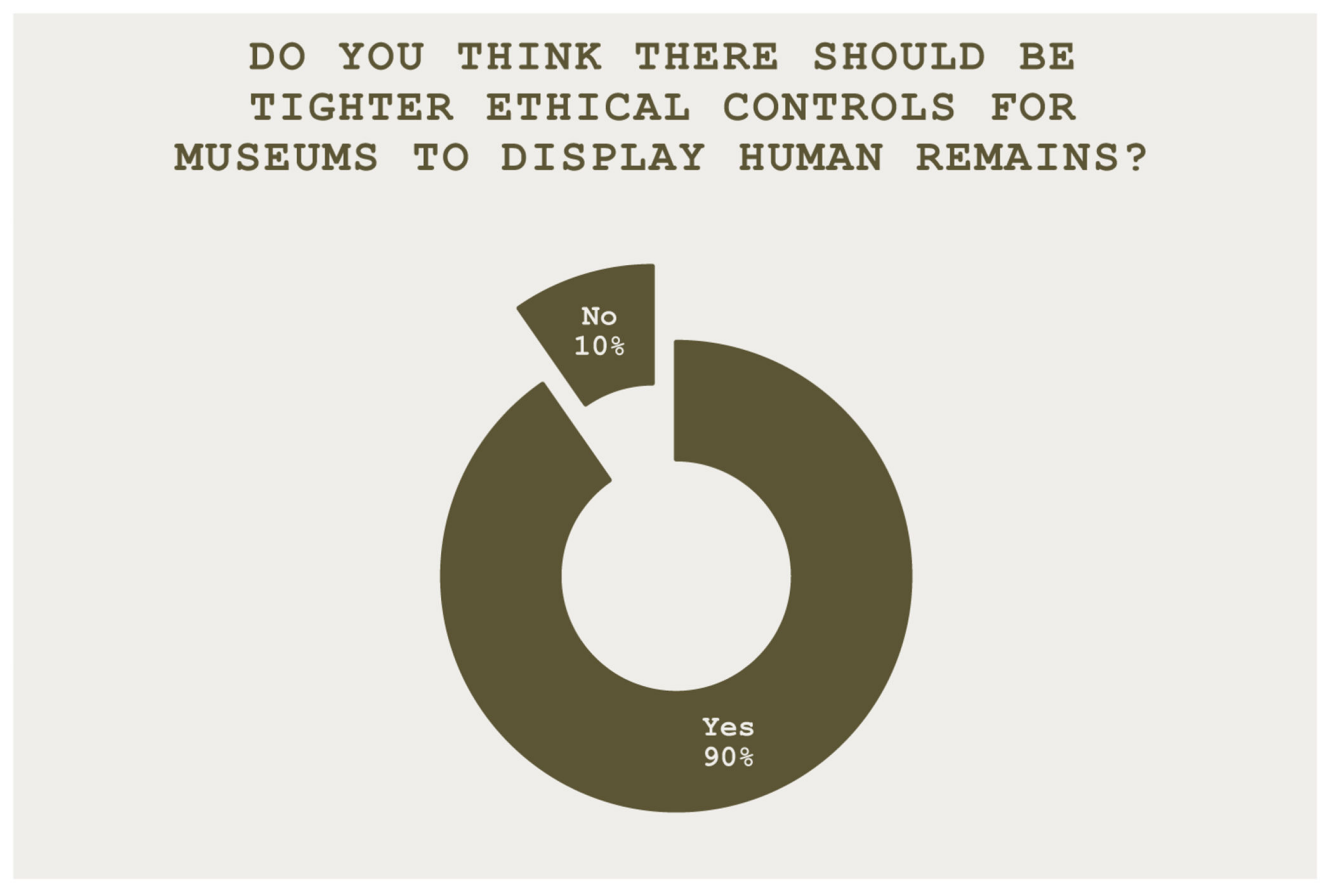
Do you think there should be tighter ethical controls for museums to display human remains? Most attendees (90%) voted yes, with 10% voting no.

The trial was presided over by one of the teams performing the role of a judge. The trial considered the question: *Human remains on trial* – *Is it ever ethical for museums to display human remains?* To begin, an overview of the topic was introduced. Then, before the trial began, attendees were invited to vote on the questions in Figures 1 and 2.

Attendees were also asked:

*What kind of emotions do you feel in relation to these kinds of human remains being held and displayed in museums?*

They had the opportunity to respond to specific examples, including:

- *Prehistorical palaeontological human remains of unknown persons*
- *Bronze Age European or ancient Egyptian/African human remains of unknown persons*
- *Anonymous European persons from the Medieval and Renaissance periods*
- *Eighteenth/nineteenth up to early twentieth century human remains (global or European) of known persons*
- *World War II up to the present-day human remains of known persons*

Arguments for the “defense” and “prosecution” were presented by two senior academics who work in museum studies. Each set out their case before calling on a range of expert witnesses. These presented provocations on the case for and against various positions and policies in the sector. An observer, also part of the project team, watched proceedings.

**Figure 3:**
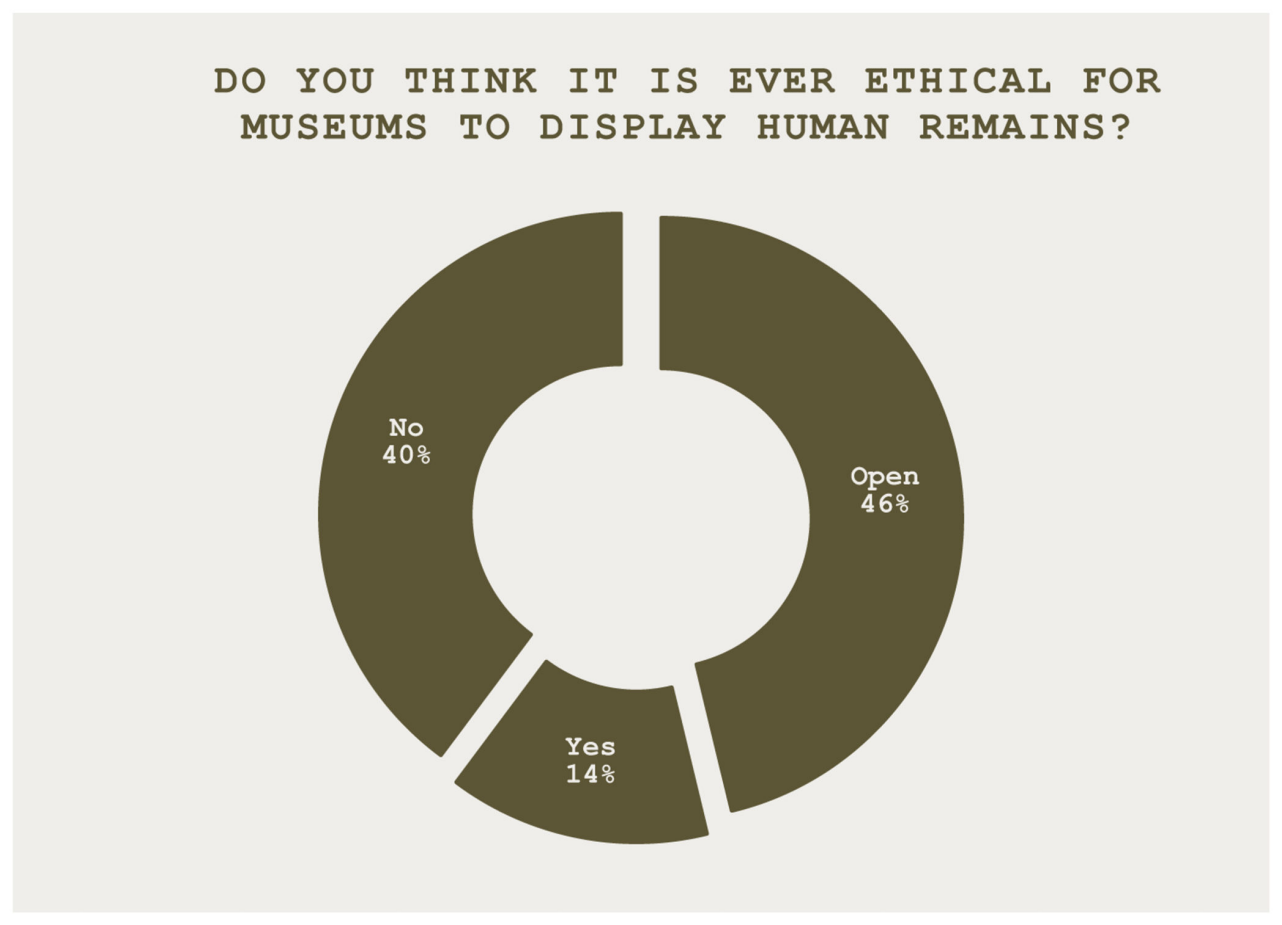
Do you think it is ever ethical for museums to display human remains? The results indicate a shift in position with less agreement (46%), increased disagreement (40%), and some (14%) taking a neutral position.

Following cross-examination of expert speakers by the prosecution and defense, attendees were invited to vote again on the question in Figure 3.

The attendees were also asked again:

*What kind of emotions do you feel in relation to these kinds of human remains being held and displayed in museums?* (see preceding list)

Data generated from the responses indicated that discomfort increases the more recent the ancestral remains are dated. Participants felt that processes of consent for collecting and displaying remains are more of a concern for ancestral remains dated after the eighteenth century. Anonymous European Medieval and Renaissance ancestral remains were rated as least valuable or necessary to display compared to other categories presented, such as World War II to present day.

The “judge” then summed up, asked the symposium to vote, and delivered the verdict, which was declared to be open. Priority actions were identified with the participants; these included further research and investigation on the provenance of remains, the sharing of good practice on the disposal or repatriation of remains, developing a remains common practice for museums and network associated with this, and adherence to professional codes of practice.

## Museums Association Conference

A panel discussion and interactive workshop took place at the annual Museums Association Conference in 2024. This session focused on ethics in the museum sector and reviewing ethics policies. This was prompted by geopolitical events such as the Covid-19 pandemic, the Black Lives Matter movement, and the humanitarian crisis in Gaza.

**Figure 4:**
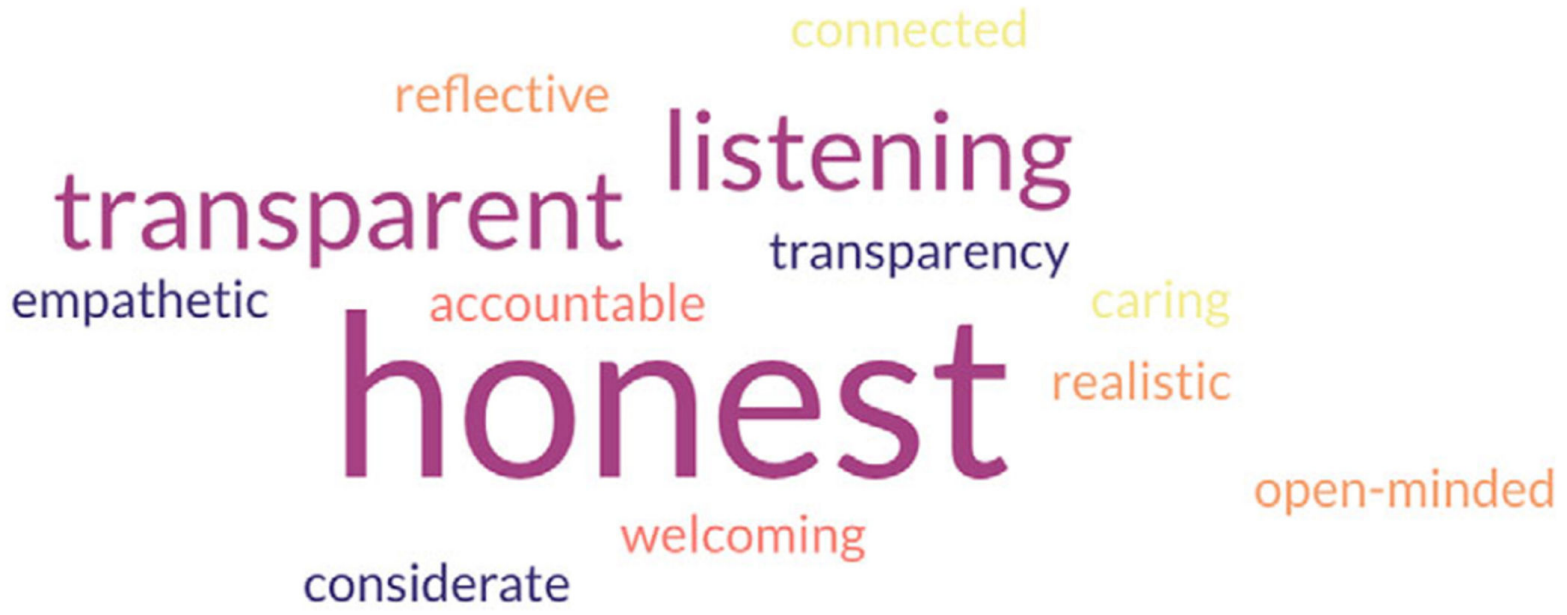
A word cloud representing responses to the prompt: “An ethical museum is …”

First, approximately 80 attendees were asked to share three words that describe what an ethical museum would look like. Key issues identified included honesty, transparency, and listening (see Figure 4). They were then asked whether museums should be spaces to address social justice. Most agreed that they should be. Finally, the historical concept of *mouseion* (derived from the ancient Library of Alexandria^27^) was presented, and its contemporary relevance was raised, namely, are museums spaces for the exchange of knowledge and debate about topics such as decolonization and antiracism? The majority agreed that they were.

Second, individuals representing different organizations shared views on issues of key ethical concern in the sector as part of a panel discussion. This included a range of organizations, small and large, across different geographical areas. While it was acknowledged that antiracism and decolonization were important and timely concerns, there was variation expressed regarding prioritzation and implementation. For example, some felt the issue was urgent given recent global events and the rise of far-right political movements, while others requested additional resources and mentoring as they lack capacity to address these issues effectively.

Following the panel presentation, attendees were presented with two case studies and were asked to explore the ethical issues within both in small groups. One featured the discovery of unknown and uncatalogued ancestral remains in a collection store, the other with violent racist content identified within an artwork contained in a museum’s inventory.

A range of ethical issues were explored by the attendees in relation to the practice of museums and heritage organizations; several main dimensions emerged. First, attendees identified the importance of policy and strategic direction to establish a clear position on the retention and display of remains. They considered the role of transparency, accountability, and risk mitigation within this.

Participatory engagement was also identified as an important way to facilitate conversation between people with lived experience. Importantly, this labor from community audiences and ancestors requires renumeration. Engagement and participation need to be understood as an important part of decision-making processes while also being transparent about broader strategic goals their institutions have.

## Discussion

These two interactive formats generated valuable data on ethical issues regarding ancestral remains. Use of online voting and small group discussions facilitated exploration of ethical issues, including adversarial positions, and gathered information on sensitive and contested topics such as the collection and use of ancestral remains. A range of diverse audiences participated, and these two events indicate that attendees engage well with the process and the discussions.

At the symposium, tentative evidence of shifting attitudes to the topic was found. That is, fewer participants agreed that displaying ancestral remains was ethical after being presented with a range of ideas and positions, in this case delivered via contrasting prosecution and defense positions and provocations from expert witnesses. At the conference, the session offered a useful opportunity for stakeholders to engage with some of the complex issues being considered as part of the wider discussion about ethics in the sector.

The use of digital voting technology may support an accessible mode of participation that is relatively anonymous. This is important when expressing views about contentious or emotive topics. While not the focus of these exercises, the emotional impact of such conversations and activities on participants should be considered. This is relevant where those participating may have personal or familial links to topics considered, for example if their ancestors were impacted by war or slavery.

The team plans to use similar methods to gather data in other locations and with different populations as part of a wider research project. This may include development of case studies to support stakeholders working through the often complex process of identifying and implementing strategies that promote ethical practices in a range of sector organizations and with different and diverse materials and objects.

## Footnotes

1
Nilsson-Stutz et al. 2025

2
National Galleries Scotland 2024

3 Chechi 2023, p. 422.

4 Krasteva 2020.

5 Kendall Adams 2020.

6 Francis 2023.

7 Thomas 2023.

8 Museums Association 2025.

9 Museums Association 2024.

10 Museums Association 2025.

11 Museums Association 2025.

12 Museums Association 2025.

13
ICOM n.d.

14 ICOM 2017.

15 Bo 2025.

16 Bo 2025.

17 BABAO 2025.

18 Nilsson-Stutz 2023.

19 Nilsson-Stutz 2023.

20 Kendall Adams and Crowdy 2025.

21 Clover 2025.

22 Joyce 2021, p. 195.

23 Kendall Adams 2025.

24 ANTITHESES 2025.

25 Culture& 2025.

26
Culture& 2025

27 Staikos 2020.
